# Risk factors of coronary heart disease among medical students in King Abdulaziz University, Jeddah, Saudi Arabia

**DOI:** 10.1186/1471-2458-14-411

**Published:** 2014-04-28

**Authors:** Nahla Khamis Ibrahim, Morooj Mahnashi, Amal Al-Dhaheri, Borooj Al-Zahrani, Ebtihal Al-Wadie, Mydaa Aljabri, Rajaa Al-Shanketi, Rawiah Al-Shehri, Fatin M Al-Sayes, Jamil Bashawri

**Affiliations:** 1 Family and Community Medicine Department, Faculty of Medicine King Abdulaziz University, Jeddah, Saudi Arabia; 2 Epidemiology Department, High Institute of Public Health, Alexandria University, Alexandria, Egypt; 3 Sixth Year Medical Student, Faculty of Medicine, King Abdulaziz University, Jeddah, Saudia Arabia; 4 Internal Medicine Department, Faculty of Medicine, King Abdulaziz University, Jeddah, Saudi Arabia

**Keywords:** Risk factors, Coronary heart diseases, Young adults, Framingham risk score

## Abstract

**Background:**

Nowadays, Cardiovascular Diseases (CVDs) represents an escalating worldwide public health problem. Providing consistent data on the magnitude and risk factors of CVDs among young population will help in controlling the risks and avoiding their consequences.

**Objective:**

The objective was to estimate the prevalence of risk factors of Coronary Heart Disease (CHD) among medical students during their clinical clerkship (4th - 6th years).

**Methods:**

A cross-sectional study was done during the educational year 2012–2013 at King Abdulaziz University (KAU), Jeddah. Ethical standards were followed and a multistage stratified random sample method was used for selection of 214 medical students. Data was collected through an interviewing questionnaire, measurements and laboratory investigations. Both descriptive and analytical statistics were done by SPSS version 21. CHD risk percent in thirty years was calculated using Framingham algorithm for each student, then the risk among all students was determined.

**Results:**

The commonest risk factors of CHDs were daily intake of high fat diet (73.4%), physical inactivity (57.9%), overweight/or obesity (31.2%) and daily consumption of fast food (13.1%). Hyper-cholesterolemia (17.2%) and hypertension (9.3%) were also prevalent risk factors. Smoking prevalence was low (2.8%). Males had significantly higher mean scores for most of CHD risk factors compared to females (*p* < 0.05). Systolic Blood pressure was higher among males (119.47 ± 11.17) compared to females (112.26 ± 9.06). A highly statistical significant difference was present (Students’t test = 4.74, *p* < 0.001). Framingham Risk Score revealed that CHD risk percent in thirty-years among all students was 10.7%, 2.3% and 0.5% for mild, moderate and severe risk, respectively.

**Conclusion:**

An alarmingly high prevalence of CHD risk factors was prevailed among medical students, especially among males. However, a low prevalence of smoking may indicate the success of “Smoke-free Campus” program. Screening risk factors of CHD among medical students and implementation of intervention programs are recommended. Programs to raise awareness about CHD risk factors, encourage young adult students to adopt a healthy dietary behavior and promote physical exercise should be initiated.

## Background

Non-Communicable Diseases (NCDs) are on continuous rise worldwide [1]. Furthermore, developing countries is experiencing a double burden of diseases; both Communicable Diseases (CDs) and NCDs [2]. It is estimated that in the developing countries NCDs will account for seven of each ten deaths by the year 2020. Among NCDs, Cardiovascular Diseases (CVDs) are the leading cause of morbidity, disability and mortality worldwide [1].

The global rise in CVDs is driven by both urbanization and its related lifestyle modifications [3]. The Kingdom of Saudi Arabia (KSA) is experiencing an alarming rising in incidence and death rates from CVDs [3-5]. A study done in the Eastern region of KSA revealed that 26% of total deaths were attributed to CVDs (27% of deaths of males and 23.5% of females) [5]. It is expected that the burden of CVDs will continue to grow in KSA due to continuous exposure to risk factors. This increase is also considering the young population; as about 60% of the Saudi population was less than 30 years [4].

Coronary Heart Disease (CHD) is the commonest cause of death from CVDs. In addition, it is one of the leading causes of disease burden [5]. Identification of risk factors contributing to the incidence of CHD is one of the major achievements of epidemiology in the 20^th^ century [6] Smoking, hypertension, diabetes mellitus, high dietary fat intake, and lack of physical exercise have been documented as independent risk factors for the development of CHD [7].

Risk factor profiles in young adulthood (18–24 years) strongly predict long-term CHD risk [8]. Understanding the magnitude and types of CHD risk factors among young adults is an important aspect in establishing targeted intervention, before disease progression occurs, through promoting lifestyle changes [7,8]. Despite these evidences, risk assessment and disease prevention efforts are lacking in this age group. Most of young adults are not screened and are not aware of their CHD risk. This leads to underestimation of the risk in spite of its high prevalence [8]. Hence, the prevalence of CHD risk factors of among young adults needs to be urgently addressed. Risk prediction algorithms have been used to detect persons at high risk for developing CVD and to pick individuals who need intensive preventive interventions. Framingham-based equations have been the most extensively used equations for clinical practice guidelines [9]. Despite these facts, limited number of studies has been conducted on estimating the prevalence of CHD risk factors among young adults in Saudi Arabia [7]. There is also lack of studies using the Framingham algorithm for CHD risk assessment. Furthermore, the American Heart Association’s 2013 recommended that screenings should include assessment of all CHD risk factors including lifestyle habits (diet, exercise, and smoking), blood pressure, glucose, and Body Mass Index (BMI) in addition to the traditional lipid panel [10]. However, most of the conducted studies in the Saudi Arabia lacked of some of the recommended items [7]. In addition, scanty studies conducted for CHD risk assessment among medical students in Jeddah. So, such studies are urgently needed.

The objective of the current study was to estimate the prevalence of risk factors of Coronary Heart Diseases among medical students, during their clinical clerkship years, at King Abdulaziz University (KAU), Jeddah.

## Methods

“Ethical statement: the study was approved by the Institutional Review Board (IRB) of the King Abdulaziz University Hospital (KAUH). The whole study was conformed to the ethical standards of the Helsinki Declaration”. A written consent was taken from each participant upon his/her acceptance to participate in the study. In addition all administrative approvals were taken.

A cross-sectional study was done during the Fifth Year Survey Elective Module of the Family and Community Medicine in the educational year 2012/2013. The study population was the medical students enrolled in their clinical clerkship years (4th - 6th) in King Abdulaziz University (KAU).

A multistage stratified random sample method was used. A sample frame was constructed and contained information on the stratification variables according to gender and grade of medical students target population. The first stratification phase was done according to gender. Then the second stratification phase was done according to their grades. The male and female leaders of each of the three grades invited and encouraged students to participate in the study. Among the selected subjects, the response rate was about 60%, with a higher response rate among females compared to males. The cause of this low response rate may be because the study included taking of a fasting blood sample from participants.

The sample size was calculated using the following formula [11]:

$$“n=\frac{z^{2}\times p\times q}{d^{2}}”$$

*n*: the minimum sample size, z = constant (1.96), *p* is the prevalence of CVDs risk factors, q = (1-p), Z is the standard normal deviation of 1.96 which correspond to the 95% confidence interval and d is the desired degree of accuracy.

As the exact prevalence of CVDs risk factors among young adults in Jeddah is unknown, so, the prevalence (p) = (q) was considered 50% (the most conservative assumption) and d was set at 0.05 to tolerate a 5% error. The calculated sample size was 196 students and it was increased during the field work to reach 214 for stratification purpose.

Each student accepted to participate in the study and signed the written informed consent was requested to come to the General Clinic of KAUH, fasting for at least 12 hours, on the next day.

### Data was collected through data collection sheet included

I. Questionnaire: An anonymous, confidential and self-administered questionnaire was used to collect:

➣Personal and socio-demographic characteristics (gender, educational year, family income and parents’ education and job).

➣History of use of drug for treatment of a chronic condition.

➣Risk factors of CHD as nutritional factors (frequency of eating foods rich in saturated fat or fast foods, frequency of eating vegetables and fruits/week), smoking habits, physical activity (regular practice of physical exercise, number of times/week and the duration of practice), time spending in TV watching or using computer.

II. Measurements: After completing the questionnaire, measurements were taken as described by D’Agostino, et al., 2008 [12] and included:

➣Weight and height: both were obtained from a lightly clothed student.

➣Blood pressure (BP) measurement: It was done while the student in the sitting position after 4 minute of rest. Systolic and diastolic blood pressure was identified at the beginning of the first and the fifth phase of the Korotkoff sounds using a mercury sphygmomanometer applying the appropriate cuff on the right arm [12].

III. Laboratory investigations: Blood sample was obtained from each participant from the antecubital vein after 12 hours of fasting. It was taken from the antecubital vein while the student in the sitting position. The biochemical evaluation was performed in the laboratory of KAUH and following the criteria of the World Health Organization Lipid Reference Laboratories. Upon arrival, the samples were centrifuged to obtain the plasma Levels of total cholesterol (TC), glucose and triglycerides (TG). They were measured by a chromatometric enzymatic method [12].

### Statistical analysis

Data was coded and analyzed using Statistical package for Social Science (SPSS) version 21 (SPSS Inc, Chicago, Ill., USA). The following statistical calculations were done to classify:

➣Body Mass Index (BMI): it was calculated by dividing weight in kilograms by the square of height in meters. BMI was then divided into normal (< 25), overweight (25 - < 30), and obese (≥ 30) [13].

➣Hypertension: classification was based on the recommendations of “The Seventh Report of the Joint National Committee on Prevention, Detection, Evaluation and Treatment of High Blood Pressure (JNC-7). It was defined as Systolic Blood Pressure (SBP) >/=140 mmHg and/or Diastolic Blood Pressure (DBP) >/=90 or concurrent use of antihypertensive agents” [14].

➣Fasting plasma glucose was classified according to the WHO classification into:

1. Normal fasting blood sugar: level plasma glucose is < 110mg/dl.

2. Impaired fasting glucose (IFG) and hyperglycemia: fasting blood glucose is ≥ 110 mg/dl or history of treatment from diabetes [14].

➣Dyslipidemias were defined according to the USA National Cholesterol Education Program (NCEP) criteria high in Low Density Lipoprotein (LDL) cholesterol as 130 mg%, hyper-tyriglyceridemia as 150 mg/dl and low in High Density Lipoprotein (HDL) cholesterol as < 40 mg/dl in males and < 50 mg/dl in females. High total cholesterol: HDL ratio was defined as > 4.5 [15,16].

The CHD full risk percent in thirty years was calculated using the Framingham’s algorithm based on BMI. The calculation of CHD was not done according to lipid profile as there were some missed cases in the triglycerides analysis. CHD risk assessment data of each student (sex, age, SBP, use of antihypertensive treatment, smoking, diabetes mellitus, BMI) was entered separately, case by case, on the web-site excel sheet using the calculator of the Framingham algorithm which developed according to Pencina and D’Agostino, 2009 [6]. Then the estimated risks of each subject entered into the SPSS data file. CHD risk of all subjects was calculated by SPSS. Stratified analysis was used to compare between risk among males and females. The 30-year risk model offered excellent discrimination (cross-validated C statistic 0.803; 95% CI, 0.786 to 0.820; internally validated C statistic 0.802; 95% CI, 0.772 to 0.832) and calibration [9]. The Framingham Risk Score has been validated in the USA, both in men and women, both in European Americans and African American. While several studies have claimed to improve the score, there is little evidence for any improved prediction beyond Framingham risk score. Based on this Score, subjects were stratified in 5 risk classes (< 5% low-risk; 5- < 10% mild-risk; 10- < 20% moderate-risk; 20- < 40% high-risk; > = 40% very high-risk)” [6].

Descriptive and analytical statistics were conducted. Chi-square test was used for comparison between two categorical variables. Student’s test was performed to compare between two independent means. A “*p* < 0.05” was considered statistically significant.

## Results

The study population was composed of 214 medical students whose age ranged from 20–28 years with a mean of 20.09 ± 1.0. Table 1 shows personal and socio-demographic characteristics of medical students enrolled in the study. Females represented about three-quarters (75.2 (% of the sample. Regarding the educational year, 35.5%, 39.3% and 25.2% of students were enrolled in the fourth, fifth and sixth year, respectively. Concerning family income, 83.5% earn more than 10,000 Saudi Riyals. About two-thirds (69.6%) of students’ fathers and 60.7% of mothers have a university degree and above.

**Table 1:** “Personal and socio-demographic” data of the sample medical students

**Variable**	**No.**	**Percent**
**Gender:**		
Male	53	24.8
Female	161	75.2
**The student’s grade:**		
Fourth year	76	35.5
Fifth year	84	39.3
Sixth year	54	25.2
**Father’s education**		
University and above	149	69.6
Less than university	65	30.4
**Mother’s education**		
University and above	130	60.7
Less than university	84	39.3
**Father’s occupation**		
Professional	151	71.2
Non professional	61	28.7
**Mother occupation**		
Professional	84	39.3
Non professional and housewife	130	60.8
Family income*		
Less than 5000	7	3.3
5000-10000	28	13.2
More than 10,000	177	83.5

*2 students refused to answer about their family income.

Concerning past history of diseases, 8.9%, 8.4% and 0.5% of medical students reported having hypercholesterolemia, hypertension, and diabetes, respectively.

The prevalence of habitual risk factors is illustrated in Table 2. It is apparent from the table that 57.9% of medical students do not practice physical exercise. Daily eating of food rich in fat and fast food is prevalent among 73.4% and13.1% of students, respectively. On the other hand, about three-quarters (76.6%) and two-fifths (38.3%) of students eat fruits and vegetables weekly. Furthermore, more than half (53.2%) of the students use computers more than 14 hours per week while 10.7% watched TV for the same duration weekly. Only a small percentage of students (2.8%) reported being current smokers.

**Table 2:** “Coronary Heat Diseases risk factors” among medical students according to their habits

**Variable**	**No.**	**Percent**
**Frequency of fruits intake**		
Never	12	5.6
Daily	38	17.8
Weekly	164	76.6
**Frequency of vegetables intake**		
Never	5	2.3
Daily	127	59.3
Weekly	82	38.3
**Frequency of intake of foods rich in fats**		
Never	6	2.8
Daily	157	73.4
Weekly	51	23.8
**Frequency of fast foods intake**		
Never	12	5.6
Daily	28	13.1
Weekly	174	81.3
**Frequency of soft drinks intake**		
Never	62	29
Daily	45	21
Weekly	107	50
**Smoking**		
Yes, current	6	2.8
Former smoker	5	2.3
Never	203	94.9
**Practice exercise**		
Yes	90	42.1
No	124	57.9
**Hours spent watching TV weekly**		
Do not watch	77	36.0
Less than 14 hours	114	53.3
More than 14 hours	23	10.7
**Hours spent on computer weekly**		
Do not use it	7	3.3
Less than 14 hours	93	43.5
More than 14 hours	114	53.2

Table 3 demonstrates prevalence of CHD risk factors among medical students according to measurements and laboratory results. About one-third of students (31.8%) weighed above the normal; 19.1% are overweight and 12.7% are obese. Hypercholesterolemia was detected among 17.2% and a similar percentage of students (16.0%) had a high level of LDL. The prevalence of hypertension according to JNC-7 classification was 9.3%; 3.7% and 7.9% of students had high systolic and diastolic blood pressure, respectively. About 97.9% of students had normal fasting blood glucose level, while 2.1% had high fasting blood glucose level (Impaired fasting glucose and hyperglycemia).

**Table 3:** Coronary heart diseases risk factors among medical students according to measurements and laboratory investigations

**Percent**	**Number**	**Variable**
**BMI**		
68.2	146	Normal
19.1	41	Overweight
12.7	27	Obese
**Systolic blood pressure**		
96.3	206	Normal
3.7	8	High
**Diastolic blood pressure**		
92.1	197	Normal
7.9	17	High
**Systolic and/or Diastolic hypertension**		
90.7	194	Normal
9.3	20	Hypertension
Fasting blood sugar*		
97.9	184	Normal fasting level
2.1	4	High fasting level
**Cholesterol**		
82.8	177	Normal
17.2	37	High
**Low density lipoprotein**		
84.0	180	Normal
16.0	34	High
Triglyceride**		
91.1	175	Normal
8.9	17	High for age

*26 missed cases for fasting blood sugar.

**22 missed cases for triglycerides.

**Figure 1:**
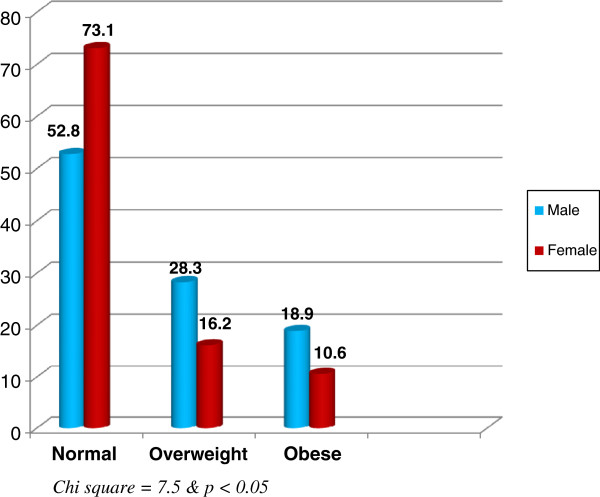
Relationship between gender and Body Mass Index among clinical years medical student in King Abdul-Aziz University.

Figure 1 demonstrates that males have higher rates of overweight and obesity compared to females. It is apparent from the figure that only about one-half (52.8%) of males had normal weight, while 28.3% and 18.9% were overweight and obese, respectively. The corresponding rates for females were 16.2% and 10.6%, respectively. A statistical significant difference was present (*X*^*2*^ = 7.54, *p* < 0.01).

Analysis of our results shows that the prevalence of hypertension was much higher among males (20.8%) compared to females (5.8%). A highly statistical significant difference was present (*X*^*2*^ = 10.82, *p* < 0.01).

Table 4 shows that that male students had higher mean levels of most of measurements compared to females. The calculated mean of BMI for males (26.27 ± 6.10) was higher than that of the females (23.30 ± 4.631). A highly statistical significant difference was found (Student’s t- test =3.72, *p* < 0.001). The mean of SBP, DBP, TGs, total: HDL cholesterol were also higher among males compared to females. Highly statistical significant differences were present. On the other hand, the mean of protective HDL in mmol/l was higher for females (1.60 ± 0.40) compared to males (1.23 ± 0.29). The results also showed that 18.5% of males and 14.0% of females had low HDL below the cutoff values for both genders.

**Table 4:** Comparison of means of anthropometric and laboratory parameters among male and female medical students

**Variables**	**Male (Mean ± SD)**	**Female (Mean ± SD)**	**Student’s t- test**	***p***
**Weight (kg)**	79.59 ± 21.02	58.43 ± 12.86	8.75	0.000
**Height (m)**	1.74 ± .068	1.57 ± 0.13	8.39	0.000
**Body mass index**	26.27 ± 6.10	23.30 ± 4.631	3.72	0.000
**Waist circumference (cm)**	92.18 ± 15.65	72.99 ± 9.06	10.99	0.000
**SBP (mm Hg)**	119.47 ± 11.17	112.26 ± 9.06	4.74	0.000
**DBP (mm Hg)**	78.55 ± 8.47	73.54 ± 9.33	3.47	0.001
**Total cholesterol (mmol/l)**	4.33 ± 0.80	4.53 ± 0.79	1.44	0.152
**Triglyceride (mmol/l)**	0.84 ± 0.32	0.67 ± 0.29	3.35	0.001
**LDL (mmol/l)**	4.97 ± 0.68	4.96 ± 0.89	1.11	0.155
**HDL (mmol/l)**	1.23 ± 0.29	1.60 ± 0.40	4.42	0.000
**Total HDL**	3.71 ± 090	2.98 ± 0.74	4.52	0.000

Using Framingham algorithm revealed that CHD risk stratified lifetime full risk percent in thirty years based on BMI among total students was 10.7%, 2.3% and 0.5% for mild, moderate and severe risk, respectively. It is much higher among males compared to females (moderate and severe risk among males was 9.4% and 1.9%, respectively) (Table 5).

**Table 5:** Framingham stratified lifetime risk Score percent of Coronary Heart Diseases in thirty years among medical students in King Abdulaziz University

**Risk class**	**Males**	**Females**	**Total**
**< 5% (Low risk)**	58.5%	95.7%	86.4%
**5%- 9.9% (Mild- risk)**	30.2%	4.3%	10.7%
**10- 19.9 (Moderate- risk)**	9.4%	0.0%	2.3%
**20-39.9 (High- risk)**	1.9%	0.0%	0.5%

## Discussion

As to our best knowledge the current study is the first study looks at CHD risk factors among young adult population in Jeddah using the Framingham algorithm to calculate the 30- years predicted risk of CHD. It may be also the first study used the recommendation of the American Heart Association’s 2013 for CHD risk assessment. All CHD risk factors including lifestyle habits (diet, exercise, and smoking), BP, glucose, and BMI in addition to the traditional lipid panel were assessed. Students were screened and told about their measurement and investigations and given the appropriate recommendations.

About 90% of individuals with CHD have at least one risk factor as smoking, diabetes, hypertension and/or hypercholesterolemia [17]. Furthermore, the US National Health and Nutrition Examination Surveys (NHANES) data among young adults aged 20–45 years (1999–2006) revealed that two-thirds have at least one CVD risk factor [10]. Nowadays, overweight and obesity are recognized as a rising pandemic [18]. The current study revealed that about one-third (31.8%) of all medical students were either overweight (19.1%) or obese (12.7%). These findings concur with results of Burke, et al. from USA who reported a similar rate of overweight/obesity (33%) among college students in University of New Hampshire’s in 2009 [19].

In the present study, the prevalence of overweight or obesity was 26.8% among females. A similar prevalence (29.1%) was reported before 2012, among females from 4 colleges of Dammam University, KSA [20].

Our work revealed a higher prevalence of overweight and obesity among male (47.2%) compared to females (26.8%). Similarly, a study conducted at the School of Medicine, Crete University; as their corresponding rates were 40% and 23%, for males and females, respectively [18]. Burke, et al. reported also that males had a higher BMI compared to females [19]. Furthermore, the rate of overweight and obesity among males in the present work coincides with results of other Saudi studies. Sabra, et al. reported a very similar rate (47.1%) among male medical students in King Fahd University in Dammam city, KSA [7]. Comparable rates were also reported from two other Saudi studies one done among male medical students in Qaseem University (46.5%) [21] and the other study was done among male and female medical students in Tibia University (44.8%) [22]. Higher rates of overweight (31%) and obesity (23.3%) were reported among male students at King Saud University, Riyadh, KSA [23]. These alarming high rates of overweight and obesity among Saudi young adults, especially males, may require rapid targeted university intervention.

On the other hand, the rates of the present study are much higher than that reported among male medical students from USA, 1999, as only one-fifth of the participants were either overweight or obese [24]. The cause of discrepancy between the current study and the USA study may be attributed to the outcome of the USA health promotion programs or due to the older time of conduction of the USA study.

Our study showed that nutritional risk factor of CHD was apparent. This is apparent from high students’ daily intake of fat- rich foods (73.4%) and fast-foods (13.1%). Meanwhile, there was low intake of healthy diet as vegetables and fruits. Sabra, et al. found also that 20.1% of medical male students were consuming fast foods in a frequency of 6–10 times/week [7]. Similarly, Larson, et al., 2008, found that 24% of male and 21% of female adolescents in Minnesota reported frequent intake of fast food (≥ 3 times/week) and these rates increased during the young adulthood [25]. In their other newer study, 2011, they reported also that frequent away-from-home fast food eating is associated with higher daily energy intake, poorer diet quality, and greater weight gain [26].

Regular practicing of physical activity provides significant benefits in reducing morbidity and mortality from CHD [7]. However, our results showed that the prevalence of non-practicing physical exercise was high (57.9%). Sedentary behaviors as playing computer games and watching TV are reported to be associated with increased prevalence of obesity and hence risk of CHD [7,27]. From our results, it was found that 10.7% and 53.2% of medical students spending ≥ 14 hours/week in watching TV and in computer usage, respectively. These results agree with results of Sabra, et al. [7].

The present work reported that the rate of current smoking is low (2.8%) compared to other similar studies. Sabra, et al. reported much higher rate; about 19% of male medical students were smokers [7]. The discrepancy between the current and the Dammam study may be because the current study was conducted among both male and females, with a small male sample, while the other study was conducted only among male with a higher prevalence of smoking. It may be also attributed to the success of Smoke-free Campus program implemented in KAU.

Regarding hypertension, it was found that about one-tenth (9.3%) of medical students in the present study were diagnosed as having hypertension; 3.7% as systolic and 7.9% as diastolic hypertension. This finding should be viewed with much concern because of the tendency of high BP to track into adult life, and because of the possibility of secondary hypertension in this age group. The study Sabra, et al. [7] reported higher corresponding rates of systolic and diastolic hypertension (13.8%% and 3.7%, respectively). This difference may be because their study was done only among males.

The current study revealed that the prevalence of hypertension was much higher among males (20.8%) compared to females (5.6%). This agrees with results from the Grete study [18]. An older study, 1998, conducted among black and Indian medical students of University of Natal, South Africa, reported lower rates among both sexes (2.5% and 4.2%, respectively) [28]. The cause of the discrepancy between the current study and South African study may be attributed to the time of conduction of their study or due to difference between the two study populations.

In the current study it was found that 2.1% had high fasting blood sugar level. This coincides with results of Greece study; 1.9% of male students and 0.6% of female students had high fasting glucose level [18].

Our study showed that male participants had higher levels of triglycerides while females had higher prevalence of the protective HDL (18.5% of males and 14.0% of females had low HDL below the cutoff values for both genders). These results are similar to results of Burke, et al. [19] and also with other findings from an Indian study conducted among the population aged 20–29 years [29].

Using Framingham algorithm of BMI full risk score revealed that CHD risk percent in thirty years among all students was 10.7%, 2.3% and 0.5% for mild, moderate and severe risk, respectively. This result is in line with another study conducted in the USA and found that the Framingham Risk Score was below 10% for all the Chicago Heart Association Detection Project in Industry -predicted risk among the 18 to 29 year old cohort [17].

Finally, eighty percent of heart disease can be prevented through diet and lifestyle modifications. Young adults are ideal targets for prevention efforts because they are in the process of establishing lifestyle habits, which track forward into adulthood [8]. Early detection of these risk factors among Saudi young adults will be very beneficial in prevention of CHD after that.

## Conclusion

An alarmingly high prevalence of different CHD risk factors revealed among medical students in King Abdulaziz University in the current study. Males had a worse risk factor profile (BMI, triglycerides, HDL cholesterol, total: HDL cholesterol, SBP, and DBP) compared to females (*p* < 0.05). Among the study population, the commonest risk factors of CHDs were daily intake of high fat diet (73.4%), physical inactivity (57.9%), overweight/or obesity (31.2%) and daily consumption of fast food (13.1%). Hyper-cholesterolemia (17.2%) and hypertension (9.3%) were also prevalent risk factors. Using Framingham Risk Score revealed that CHD risk percent in thirty years among all students was 10.7%, 2.3% and 0.5% for mild, moderate and severe risk, respectively. It is much higher among males compared to females.

Implementation of multi-factorial CHD risk screening among Saudi medical students and young adults, and application of intervention programs for those at higher risk is highly recommended. Educational programs to raise health awareness of medical students about CHD risk factors and to encourage them to adopt a healthy dietary behavior, promote physical exercise and smoking cessation should be initiated. Promotion of healthy active lifestyle and prevention of obesity should be a health priority. Implementing surveillance activities to monitors CHD risk factors and determinants among medical students and young adults and to identify the morbidity and mortality from CHD is recommended. Implement medical schools interventions including through regulatory and legislative actions, for the CHD related risk factors as tobacco use, unhealthy diet, lack of physical activity is also required. Further researches involving adults inside and outside the medical schools need to be done to evaluate the effect of knowledge on behavioral CHD risk factors and for better understanding of this preventable epidemic.

### Limitations of the study

The number of females was higher than the number of males as the acceptance rate among females was higher than males. Some of the finding like smoking, BMI and blood tests may be affected.

There is a limitation of applying Framingham risk score for non-western population, young population and weather the risk score was previously standardized to be used for Saudi population. On the other hand, although several studies have claimed to improve on the Framingham risk score, there is little evidence for any improved prediction beyond this score.

## Abbreviations

NCDs: Non-communicable diseases; CVDs: Cardiovascular diseases; CHD: Coronary heart disease; KSA: Kingdom of Saudi Arabia; KAU: King Abdulaziz University; IRB: Institutional review board; KAUH: King Abdulaziz University Hospital; BP: Blood pressure; SBP: Systolic blood pressure; DBP: Diastolic blood pressure; IFG: Impaired fasting glucose; LDL: Low density lipoprotein; HDL: High density lipoprotein.

## Competing interests

There are no financial or non-finical competing interests.

## Authors’ contributions

NKI: Select the study topic, construct the frame of work, construct data collection methods, conduct data analysis, supervise the whole work, write and revise the paper and submit it to the journal. Students: MM, AA, BA, EA, MA, RA, RA: Help in construction of frame of work, conduct the field work and data entry on SPSS and Framingham excel sheet, help in writing and drafting the paper. FMA: Help in construction of frame of work, help in construction of data collection methods, help in conduction of examination, facilitate conduction of laboratory analysis and help in writing the paper. JB: Help in construction of frame of work, help in construction of data collection methods, help in conduction of measurements and help in writing the paper. All authors have read and approved the final manuscript.

## Pre-publication history

The pre-publication history for this paper can be accessed here:

http://www.biomedcentral.com/1471-2458/14/411/prepub
